# Laser Microdissection of Specific Stem-Base Tissue Types from Olive Microcuttings for Isolation of High-Quality RNA

**DOI:** 10.3390/biology10030209

**Published:** 2021-03-10

**Authors:** Isabel Velada, Esther Menéndez, Rita Teresa Teixeira, Hélia Cardoso, Augusto Peixe

**Affiliations:** 1 MED—Mediterranean Institute for Agriculture, Environment and Development, Institute for Advanced Studies and Research, Universidade de Évora, Pólo da Mitra, Ap. 94, 7006-554 Évora, Portugal; esthermenendez@uevora.pt (E.M.); hcardoso@uevora.pt (H.C.); 2 BioISI—Biosystems & Integrative Sciences Institute, Faculty of Sciences, University of Lisbon, 1749-016 Lisbon, Portugal; rtteixeira@fc.ul.pt; 3 MED—Mediterranean Institute for Agriculture, Environment and Development and Departamento de Fitotecnia, Escola de Ciências e Tecnologia, Universidade de Évora, Pólo da Mitra, Ap. 94, 7006-554 Évora, Portugal; apeixe@uevora.pt

**Keywords:** cryosectioning, single cell, cortex, epidermis, phloem, adventitious roots, OCT medium, PEN-membrane glass slide, RNA quality number

## Abstract

**Simple Summary:**

Only a small portion of the stem cells participate in the process of adventitious root formation and the cells/tissues types involved in this process is species-dependent. In olive, it is still unclear which type of cells acquire competence for rooting. Regardless, the entire stem nodal segment (containing a mixture of distinct cell types) continues to be used in studies related to the molecular mechanisms underlying this process. Laser microdissection (LM) technology has been applied to isolate specific tissue and cell types. However, it is difficult to find a standard LM protocol suitable for all plant species and cell types and, thus, LM procedures must be developed and optimized for each particular tissue. In this study, we aimed to evaluate the efficiency of a LM protocol in olive microcuttings stem-base samples. This work presents a simple, rapid and efficient LM procedure for harvesting specific tissue types used for further high-quality RNA isolation. This will encourage future cell type-specific transcriptomic studies, contributing at deciphering rooting-competent cells in olive stems and to better understand the molecular mechanisms underlying the process of adventitious root formation.

**Abstract:**

Higher plants are composed of different tissue and cell types. Distinct cells host different biochemical and physiological processes which is reflected in differences in gene expression profiles, protein and metabolite levels. When omics are to be carried out, the information provided by a specific cell type can be diluted and/or masked when using a mixture of distinct cells. Thus, studies performed at the cell- and tissue-type level are gaining increasing interest. Laser microdissection (LM) technology has been used to isolate specific tissue and cell types. However, this technology faces some challenges depending on the plant species and tissue type under analysis. Here, we show for the first time a LM protocol that proved to be efficient for harvesting specific tissue types (phloem, cortex and epidermis) from olive stem nodal segments and obtaining RNA of high quality. This is important for future transcriptomic studies to identify rooting-competent cells. Here, nodal segments were flash-frozen in liquid nitrogen-cooled isopentane and cryosectioned. Albeit the lack of any fixatives used to preserve samples’ anatomy, cryosectioned sections showed tissues with high morphological integrity which was comparable with that obtained with the paraffin-embedding method. Cells from the phloem, cortex and epidermis could be easily distinguished and efficiently harvested by LM. Total RNA isolated from these tissues exhibited high quality with RNA Quality Numbers (determined by a Fragment Analyzer System) ranging between 8.1 and 9.9. This work presents a simple, rapid and efficient LM procedure for harvesting specific tissue types of olive stems and obtaining high-quality RNA.

## 1. Introduction

Higher plants are composed by a myriad of tissues and hence, of different cell types. Distinct cell types are likely to host different biochemical and physiological processes being accompanied also by changes in gene expression profiles, protein and metabolite levels [1,2]. Therefore, in order to tackle the differences in cellular responses, studies which make use of the whole tissue, or even the entire plant organ, are being replaced by techniques performed at the cell- and tissue-type level because it is well accepted that, specific information given by a particular cell can be masked when using a mixture of distinct cells [3,4,5,6,7,8,9]. Studies performed at the cell and tissue level contribute to distinguish the information given by different cell types, it also helps to detect low-abundance molecules [10], allowing the identification, with more accuracy, of the function/specialization of each cell type and tissue. Therefore, cell type-specific studies can provide a detailed spatiotemporal characterization of distinct cell types present in plants, their developmental trajectories, and their transcriptional regulatory pathways [8].

Laser microdissection (LM) technology has been used to isolate specific tissue and cell types from complex tissue samples. This method utilizes a light microscope (with the capacity to use brightfield, darkfield and fluorescence) fitted with a laser device which allows the cutting of the area of interest and further harvesting of the microdissected cells. This technology uses one of two systems; in laser capture microdissection (LCM), developed by Arcturus^®^ (https://www.leica-microsystems.com (accessed on 30 June 2020)), target cells are harvested from tissue section by bonding them to a plastic film at sites activated by a near-infrared (IR) laser. In laser cutting microdissection, target cells are first cut free from the tissue section by an UV laser and collected by one of several methods. One of the methods is the laser pressure catapulting (LPC) method (developed by PALM-Microlaser Technologies GmbH., Bernried, Germany), in which the target area is cut free with an UV nitrogen gas laser and then catapulted toward a collection vessel. In one other method, the laser microdissection (LMD) developed by Leica Microsystems, the biological section is in an inverted orientation, and the UV-circumscribed target is collected in a tube by gravity (reviewed by [11]).

Although LM techniques have been applied in animal research for a long time now [12], its application in plant cells came a bit later. In fact, it was only in 2002 that the use of this technique in plant tissues was first reported by Asano et al. [13] in a study of phloem-specific gene expression in rice. This happened, mostly because microdissection of plant tissues and individual cells poses a challenge due to the presence of a cell wall, a large central vacuole present in most fully differentiated cells and, in some tissues, to large intercellular spaces [14,15,16]. Despite such constrains, LM applied to plants [17] has been optimized and today, this technique has successfully been applied on different research areas of plant biology. A relatively high number of studies have been reported so far on transcriptomic, proteomic and metabolomic analysis, mainly focused on individual plant tissue and cell types, with various aims, such as to study cell specialization for growth and development, protection and stress responses [18,19,20,21,22], to quantify phytohormones at a tissue level [23], to understand the network of transcriptional regulators controlling processes like inflorescence development and fruit development and ripening [10,24,25], or to study the embryogenesis process [26]. Worthy of note is the fact that in all these works there is not an available standard protocol suitable for all plant species and cell types, implying that LM protocols must be developed and optimized for each tissue type.

Regarding root development, there are few studies focusing on specific tissue and cell types and the ones that exist are mainly related to lateral root formation ([27,28]). Our research group has been focused on the study of the molecular mechanisms underlying adventitious root (AR) formation in olive [29,30,31,32,33,34]. Adventitious roots may be formed from stem, leaves and non-pericycle tissues [35]. The anatomical structure of the olive stem-base, at the site of adventitious root formation, is organized from the outermost to the innermost rings as: epidermis, hypodermis, cortex, phloem, cambium, xylem and the pith tissues. The cambial zone consists of few layers of flat cells between the xylem and the phloem; the epidermis is formed by one or two cell layers; and the cortex is constituted by several layers of large parenchymatous cells [29]. Although LM technology is already being widely used in other research areas, the majority of recent studies dealing with adventitious root formation still use the whole tissue of the stem [36,37,38,39], even though it is well accepted that only a few cells become competent to respond to the stimulus to develop adventitious roots. Furthermore, still today, there is no consensus regarding which type of cells acquire competence for the formation of AR. For example, in olive cuttings, regardless of the cultivar’s rooting ability, most authors have observed adventitious roots arising from the cambial region of the stem ([29]). However, Macedo et al. [29] did not observe any adventitious roots arising from this tissue in ‘Galega vulgar’ olive. These authors found that the first meristemoids developed from the upper phloem and from the cortex/sub-epidermal (hypodermis) region. 

To the best of our knowledge there are only two studies on the molecular mechanisms of AR formation working at the cellular and tissue level. Almeida et al. [40] isolated, by LM, cells from the cambium zone of eucalyptus microcuttings stem-bases to perform gene expression analysis. Also, making use of LM, Stevens et al. [41] collected cortex and phloem sections from black walnut stems for further RNA isolation and gene expression analysis, as well. Both works applied two completely different procedures for sample preparation for LM, reinforcing the idea that for each tissue type, proper sample preparation for LM are quite different. Therefore, the aim of the present work was to test the efficiency of a protocol for the preparation of olive microcuttings stem-base samples to be used in LM technology for the isolation of specific tissue types. This will pave the way for further studies on cell type-specific gene expression analysis in stem-base tissues to better understand the molecular basis of adventitious rooting process. 

## 2. Methods

### 2.1. Plant Material and Growth Conditions

**Figure 1:**
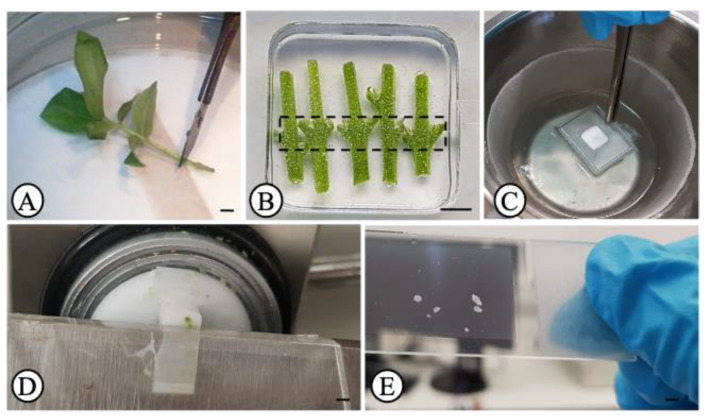
Plant material preparation for laser microdissection. (**A**) olive microcutting showing the cutting site of the stem nodal segment; (**B**) stem nodal segments in cryomolds containing OCT medium; (**C**) cryomolds containing the segments being frozen in liquid nitrogen-cooled isopentane; (**D**) cryosectioning; (**E**) cryosections on a PEN-membrane glass slide after dehydration and drying. Scale bars, 4 mm.

In vitro-cultured *Olea europaea* L. plantlets of cv. ‘Galega vulgar’ (clone 1441) [42], in a developmental phase normally used for rooting experiments [30,31,32], were utilized here to collect distinct cellular types from the stem node sections such as; epidermis (plus hypodermis), cortex and phloem. Plantlets were kept in 500 mL glass flasks containing 75 mL semi-solid culture medium, in a plant growth chamber at 24 °C/21 °C (±1 °C) day/night temperatures, with a 15 h photoperiod, under cool-white fluorescent light at a photosynthetically active radiation (PAR) level of 36 μmol/m^2^ s^−2^ at culture height. The nodal segment from the basal portion (approx. 1 cm from the base) of each microcutting were sectioned (Figure 1A) and used for LM.

### 2.2. Sample Preparation

Five stem nodal segments were placed directly into cryomolds (Sakura Finetek, AJ Alphen aan den Rijn, The Netherlands) filled with Tissue-Tek optimal cutting temperature (O.C.T.) compound (Sakura Finetek) kept at 4 °C (Figure 1B). Three technical replicates (cryomolds), each containing five segments (five biological replicates further treated as a pool) were prepared. A total of fifteen segments were analyzed. Each group of five segments was collected from plantlets from a different glass flask. Cryomolds were immediately frozen with isopentane (Acros Organics, Morris Plains, NJ, USA) (to avoid ice crystal formation) [43], placed inside a metal beaker which in turn, was placed inside a styrofoam container with liquid nitrogen (Figure 1C). By the time OCT turned white, cryomolds were immediately stored at −80 °C until use. 

### 2.3. Cryosectioning

Before microdissection, the sample-containing OCT blocks were sectioned with a cryostat (Thermo Fisher Scientific, Waltham, MA, USA) (Figure 1D). Before proceeding to sectioning, the OCT blocks were left inside the precooled to −20 °C cryostat for 30 min for acclimatization. Transversal sections of 15 μm in thickness were mounted on polyethylene naphthalate (PEN)-membrane glass slides (Leica, Herborn, Germany), containing a few drops of 100% ethanol over the membrane, which were maintained on ice during cryosectioning. Before use, slides were sterilized under UV light for 30 min. Around 15–20 sections were collected from the basal stem node (usually submitted to auxin treatment for root induction trials) (Figure 1B, dashed line). The fixation and dehydration of the slides were performed according to Teixeira and co-authors [18] with some modifications. Briefly, slides were rinsed twice with 100% xylene for 30 sec each, followed by 70% (*v/v*) ethanol (in DEPC water cooled to −20 °C) for 30 s, and finally by 100% ethanol for 2 min. Slides were then air dried at room temperature for 2 min and immediately used for microdissection (Figure 1E).

### 2.4. Laser Microdissection (LM)

**Figure 2:**
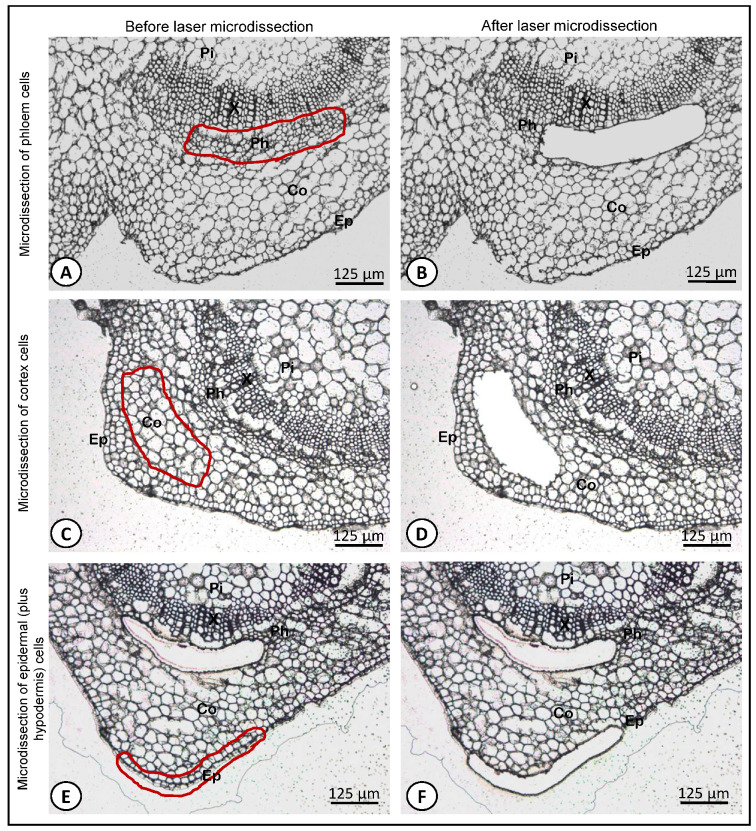
Cryosections of the stem nodal segments of olive microcuttings at the site of adventitious root formation before (**A**,**C**,**E**) and after (**B**,**D**,**F**) laser microdissection of cells from the phloem (**A**,**B**), cortex (**C**,**D**), and epidermis (plus hypodermis) (**E**,**F**) tissues. Pi, pith; Co, cortex; Ep, epidermis; Ph, phloem; X, xylem.

Microdissection was performed using a Leica LMD6500 laser microdissection system (equipped with a Leica DM6000B microscope with a 355 nm wavelength laser, pulse frequency 80 Hz, pulse length < 4 ns and max. pulse energy 70 μJ; and coupled to a Leica CC7000 camera; Leica, Microsystems, Wetzlar, Germany) with the following laser parameters: power 60, aperture 45, speed 10–30 (depending on the success in tissue removal), and specimen balance 0, using the Leica LMD software (V6.7.2.4295). Cells from the phloem (Figure 2A,B), cortex (Figure 2C,D) and epidermis (plus hypodermis) (Figure 2E,F) were microdissected and harvested separately. The area given by the LMD software (Figure S1A) corresponding to the dissected region was recorded to calculate the approximate number of harvested cells per tissue type (Figure S1B). Considering an average cell size of 20 µm, a minimum of 400,000 µm^2^ dissected area was recovered (Figure S1A), in order to obtain around a minimum of 1000 cells per sample. Microdissected cells from the same tissue type and from the same group of five biological replicates (five segments) were pooled in order to obtain a minimum of 1000 cells per tube. The microdissected regions were collected by gravity into the cap of AdhesiveCap 500 opaque microtubes (Zeiss, Hamburg, Germany). Microdissected tissues were resuspended in 65 μL RLT (containing β-mercaptoethanol) buffer from the RNeasy Micro kit (Qiagen, Hilden, Germany) plus 10 μL of Ambion^®^ Plant RNA Isolation Aid (Life Technologies Corporation, Carlsbad, CA, USA). Tubes were then closed and kept upside down on ice for 2 min and then briefly spun down in a centrifuge at 10,000 rpm for 1 min and stored at −80 °C until RNA extraction. 

### 2.5. RNA Isolation and Integrity Analysis

Total RNA was isolated with the RNeasy Micro kit (Qiagen) according to the ‘Purification of Total RNA from Microdissected Cryosections’ protocol and according to the supplier’s instructions, and eluted in 10 µL volume of RNase-free water. The concentration and integrity (by the RNA Quality Number, RQN) of total RNA were evaluated with the Fragment Analyzer Automated CE System (Advanced Analytical Technologies GmbH., Heidelberg, Germany) with the High Sensitivity RNA Analysis Kit (Advanced Analytical Technologies GmbH.), according to the manufacturer’s instructions. 

## 3. Results and Discussion

**Figure 3:**
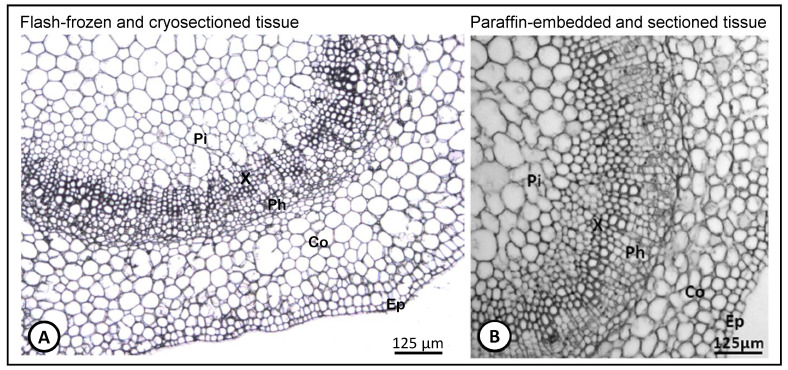
Cross sections of the stem nodal segments of olive microcuttings at the site of adventitious root formation showing a vascular bundle (Pi, pith; Co, cortex; Ep, epidermis; Ph, phloem; X, xylem). (**A**) Tissue cryosection prepared as described in the material and methods of the present work. (**B**) paraffin-embedded tissue prepared as described in Macedo et al. [29].

In order to assess the integrity of the cryosectioned olive stem-base tissues prepared as described here, we established a comparison using the same tissues but prepared by paraffin-embedding method described by Macedo et al. [29] (Figure 3). It was possible to observe that the cryosectioned tissues previously flash-frozen (Figure 3A) showed a histological morphology perfectly comparable to its paraffin embedded counterpart tissues (Figure 3B). The method applied in the present study in sample preparation for LM allows to perfectly distinguish all cell types, such as the pith, xylem, phloem, cortex and epidermis. Moreover, all cell types maintained their structure and no cell rupture was observed in any of the tissue types.

To date, there is no standard LM protocol suitable for every plant species, organ or tissue type of interest, meaning that many different procedures have been established and optimized [13,14,15,16,44,45,46,47] for RNA extraction and further gene expression analyses. The crucial point in these protocols is that there must be a balance between a good morphology and cell integrity in histological sections (so that different cell types can be easily distinguished to be correctly identified and selected) and the integrity of the molecule’s preservation to be analyzed (e.g., nucleic acids, proteins, metabolites). 

One method widely used, because it permits to get high level of histological morphology and cell integrity, is the paraffin-embedding method. Albeit paraffin-embedding protocols enabling a good structural cell maintenance, prior to embedding, samples are usually submitted to fixation procedures. Some of the fixatives used are acetone, ethanol, ethanolic acetic acid, and/or methacarn (methanol/chloroform/acetic acid) ([11]). However, it has been reported that fixatives may compromise the yield of recovered biomolecules, especially the crosslinking fixatives (e.g., aldehydes and formalin), as opposed to the precipitating fixatives (e.g., acetone and ethanol) which improve yield rates of biomolecules ([48]). Furthermore, fixation and embedding steps can be time consuming, require high tissue-handling, could prevent an uniform extractability of molecules from the tissue sample and can be also a source of contamination ([47]). For all those reasons, the method may not be suitable for gene expression analysis since, during the procedure, total RNA can be degraded by the action of RNases, compromising total RNA yield and quality. This has been demonstrated for example by Blokhina et al. [14] who found that paraffin-embedded aspen samples caused RNA degradation. In the same line, Abbott et al. [47] observed that the morphology of formalin fixed, paraffin embedded cross-sections of spruce stems was of good quality, but the RNA was degraded compared to unfixed samples. Also, Olsen and Krause [44] showed that with their method the quantitative yield of RNA was comparable between acetone-fixed and non-fixed material, however, overnight acetone fixation reduced the quality of the isolated RNA. 

Cryosectioning is one method that has also been applied for sample preparation for LM which has been reported to be a good option to obtain RNA of high quality ([48]). In fact, it has been reported that the yield and quality of RNA obtained from cryosectioned samples is superior to that derived from paraffin-embedding sections [49,50]. However, the reverse of the medal of this method is that it reduces cell morphology integrity. This is more prominent in plant tissues than in animal tissues because when fresh plant tissues are frozen, ice crystals can form in vacuoles (causing ruptures) and in the large air spaces between cells, promoting anatomical disruptions and, consequently, damaging the specimen ([11]).

To overcome the problem related to plant cell structure damage due to freezing, plant material is usually infiltrated with cryoprotection agents prior to freezing, such as sucrose [26,51]. However, sucrose concentrations ought to be optimized for each type of tissue because it has been reported that low sucrose concentrations (15%) may allow plant tissues to have good morphology, however, the obtained RNA shows some degradation. On the other hand, incubating samples directly in higher sucrose concentrations (35%) increases RNA quality, but the morphology of the sections may be strongly compromised [45]. Other disadvantages of using sucrose have been reported. For example, despite allowing preserving tissue morphology, sucrose might interfere with laser cutting [47]. Furthermore, infiltration of sucrose solutions could trigger displacement of RNAs to the neighboring cells [52]. 

A number of plant LM studies have utilized fresh-frozen, cryoembedded, and cryosectioned plant tissues [13,16,26,53,54]. Nevertheless, these studies still utilized some kind of fixative and cryoprotectant before cryosectioning. Abbott et al. [47] could observe that cryosections without any kind of cryoprotection or fixation, despite showing reduced quality of morphology, were still of enough high quality to identify specialized cell types and tissues and provide higher RNA yield and integrity. In the same line of this work, in the present study, cryosections were taken from frozen stem nodal segments of olive microcuttings without any prior fixation or cryoprotection in order to limit the time that RNases might be active, thereby avoiding RNA degradation. We employed a procedure where fresh plant material was placed rapidly and directly into cryomolds filled with OCT medium and fast-frozen in liquid nitrogen-cooled isopentane (Figure 1B,C). The use of isopentane helps to freeze the tissue in a homogeneous fashion preventing this way, the formation of ice crystals from naturally occurring water molecules within the cells [43]. The use of isopentane is widely used in animal tissues [55], but it is not commonly used in plant tissues. The application of OCT is particularly suitable for soft tissues [10,16]. 

Other aspect that ought to be optimized, is the sections thickness during cryosectioning (Figure 1D). The thicker the section, the harder it will be for the laser beam to cut plant cell walls, but very thin sections will not comprise enough cytoplasmic content forcing the pooling of a much larger number of cells. In the present work, 15 μm thick sections showed a good performance for microdissection procedure. This means that the sections showed high histological integrity and the regions were cut effectively with just a single round of the laser beam. This is very important to reduce the expose time that sections are at room temperature (avoiding the action of RNases) and also, avoiding burning/charring of the sample, in case several laser rounds and/or increments of laser intensity are to be applied. Nevertheless, one advantage of having thicker sections is that more cell content is collected from the same section hence enhancing the RNA yield and quality [44]. In this work, cryosections thinner than 15 μm were not tested because, as reported by others [14], despite the fact thinner sections are easier to cut, they are also more fragile and prone to breaking more frequently after the dehydration step. 

Once cryosectioning is complete, cryosections are normally transferred to a glass or membrane frame slide and then subjected to fixation and dehydration steps. During these steps, the integrity of the tissue can also be compromised and therefore they must be also optimized depending on the tissue type. This study used a (PEN)-membrane glass slide to mount the sections (Figure 1E). The adherence of tissue sections to the membrane is more difficult than on the glass. This issue was overcome by placing a few drops of 100% ethanol over the membrane which helped preventing the sections from slipping out of the slides. Xylene was used here as a clearing agent in order to render the tissues transparent [56] and also to remove the surrounding OCT medium from the sections. OCT medium must be completely removed because it can interfere with the proper functioning of the laser beam while microdissecting the samples. The successive passages of the slides by ethanol 70% and 100% are necessary for complete dehydration of the sections enabling a safe RNAse-free activity manipulation of the slide during microdissection, a procedure that can take up to for several hours. After the dehydration step, sections had to be completely dry out in order to allow a proper cutting by the laser during microdissection. Here, 2 min at room temperature were enough to dry the tissue sections completely. 

In the present study, applying the laser settings described in the material and methods section, it was possible to collect efficiently microdissected areas, meaning that the cut areas were easily released (Figure S2) from the surrounding tissue and the application of a single laser excision round was sufficient to allow them to fall down immediately into the collection tubes. No signals of burning/charring of the samples were observed in any of the three types of tissue analyzed here (Figure S3). Very few regions needed a second or third ablation laser round to cut the cell walls. The efficiency of the laser cut depends not only on the laser settings but also on the tissue thickness. During LM, while regions of interest are selected and cut, the slides were maintained at room temperature and therefore it is very important that this procedure takes as little time as possible to avoid RNA degradation. Abbott et al. [47] observed no detectable decrease in RNA integrity for slides left at room temperature for up to four hours. Based on those findings, and to assure that there was no loss in RNA integrity, in the present study, no tissue section took more than 1 h to be processed. 

One obvious limitation of LM, is the amount of tissue harvested. The amount of tissue isolated using LM is usually referred as to the number of collected cells. The software can estimate the number of cells collected based on the cell size of the tissue and the surface of area harvested. Plant cell size varies between different tissues and plant species, ranging from 10 to 100 μm [57]. Usually, if the amount of LM-harvested tissue is low, the amount of the corresponding isolated RNA is also limited. The amount of total RNA obtained depends on the number and size of cells acquired. As a rule, fully differentiated plant cells may be quite large and extremely vacuolated and contain far less RNA/μm^2^ than smaller, densely packed cytoplasmic cells found in undifferentiated and actively dividing tissues. Both animal and plant cells typically contain on the order of 10–100 pg of total RNA per cell, depending upon the tissue type ([11]). In this study, a minimum surface area around 400,000 μm^2^ was collected from each tissue type (Table 1). 

At the end of the RNA isolation protocol (RNeasy Micro kit, Qiagen) the resultant RNA was eluted in 10 µL volume of RNase-free water. The RNA concentration (by the Fragment Analyzer system) ranged between 0.974 ng/µL to 3.364 ng/µL, and the collected surface area ranged between 411,020 and 797,119 μm^2^ (Table 1). Olsen and Krause [44] reported that they could isolate around 7 ng total RNA from 1 mm^2^ total surface area of *Cuscuta reflexa*. By measuring *C. reflexa* cell sizes in the sections, the authors determined the average yield of total RNA per *C. reflexa* cell area of around 27 pg. Chandran et al. [58] utilized <2500 harvested plant cells that yielded between 1 and 100 ng. Nevertheless, today it’s possible to find commercial total RNA extraction and complementary DNA synthesis kits design to work with very low amounts of transcripts (around picograms). Sakai et al. [59] reported that a low amount of total RNA (i.e., 10 pg) is sufficient to generate libraries and produce RNA-seq data. These authors even recommend a minimum of 50 pg of total RNA to perform a comprehensive analysis of the expressed genome. Hence, it seems that the limited amount of RNA is becoming less of an issue ([11]), rather than the quality of RNA. 

**Figure 4:**
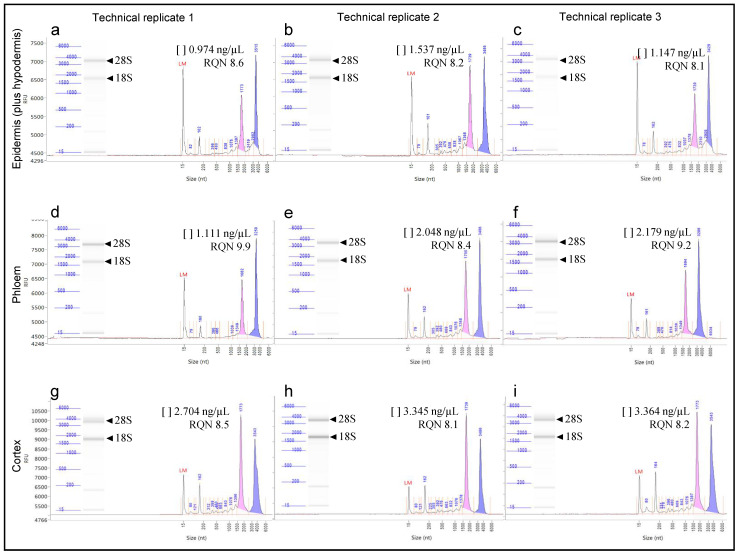
Integrity of the total RNA isolated after microdissection of the epidermis (plus hypodermis) (**a**–**c**), phloem (**d**–**f**) and cortex (**g**–**i**) from stem-base tissue cryosections, using the Fragment Analyzer system. The three graphics for each tissue corresponds to three technical replicates. The results are shown as an electropherogram showing two peaks and a digital gel showing two bands corresponding to the two ribosomal RNA subunits (18SrRNA and 28SrRNA). The concentration ([ ]) for each RNA sample is indicated as well as the corresponding RNA Quality Number (RQN).

RNA quality is usually assessed by micro-capillary electrophoresis (separation of RNAs by size), by using systems such as the Bioanalyzer (Agilent) or the Fragment Analyzer (Advanced Analytical). These systems report a number indicator of RNA quality, the RNA Integrity Number (RIN; Bioanalyzer) or RNA Quality Number (RQN; Fragment Analyzer). These numbers are based primarily on assessing the level of degradation of 18S and 28S rRNAs. An elevated threshold baseline and a low 28S:18S rRNA ratio is indicative of RNA degradation. A RIN value of 1.0 indicates total degradation of RNA, whereas a value of 10.0 suggests that the RNA is fully intact [52,60]. In the present work, the RNA quality of our samples was evaluated by the RQN (Fragment Analyzer system) in order to know whether microdissected cells could be used for subsequent gene expression studies. Total RNA isolated from all the three types of tissue tested of olive stem basal segments showed extremely high quality, demonstrated by the RQN ranging between 8.1 and 9.9 (Table 1 and Figure 4). Figure 4 shows the electropherogram and the digital gel showing the two peaks and the two bands, respectively, corresponding to the two ribosomal RNA subunits (18SrRNA and 28SrRNA) obtained from the epidermis (plus hypodermis) (Figure 4a–c), the phloem (Figure 4d–f) and the cortex (Figure 4g–i) tissues, of the three technical replicates. The total RNA obtained in technical replicates 1, 2 and 3 (each consisting of a pool of five biological replicates) from the epidermal (plus hypodermis) tissue gave a RQN of 8.6, 8.2 and 8.1, respectively. RNA from the phloem tissue showed a RQN of 9.9, 8.4 and 9.2, and from the cortical tissue, the RQN values were of 8.5, 8.1 and 8.2 for technical replicates 1, 2 and 3, respectively. 

## 4. Conclusions

This work presents a simple, rapid and efficient LM protocol for stem basal segments of olive microcuttings to isolate specific tissue types and obtain high-quality RNA. Here, there was a concern to limit as much as possible the handling of tissue sections during all procedure as well as the amount of time they stayed at room temperature, in order to avoid/minimize RNA degradation. This procedure uses flash-frozen (avoiding ice crystals formation) samples without any kind of fixation and cryoprotection. The protocol permits to obtain total RNA of high quality and in sufficient amount for further works on gene expression analysis and/or high-throughput transcriptomic analyses. As a future work, we propose to use this protocol for harvesting regions corresponding to the first cells divisions, the meristemoids structures and the morphogenic root zones observed after olive rooting induction (Figure S4) (for details see [29]), to obtain RNA of high quality to be used in cell type-specific transcriptomics studies. This will allow unraveling rooting-competent cells and to better understand the molecular mechanisms underlying the adventitious rooting process. 

## Figures and Tables

**Table 1:** Microdissected area and the corresponding yield and quality of the RNA isolated for each tissue type and per technical replicate.

Tissue Type	Technical Replicate	Microdissected Area (μm^2^)	RNA Concentration (ng/μL)	RQN ^1^
**Epidermis** **(plus hypodermis)**	1	411,020	0.974	8.6
	2	573,546	1.537	8.2
	3	513,634	1.147	8.1
**Phloem**	1	651,364	1.111	9.9
	2	648,912	2.048	8.4
	3	797,119	2.179	9.2
**Cortex**	1	669,198	2.704	8.5
	2	769,165	3.345	8.1
	3	781,354	3.364	8.2

^1^ RQN: RNA Quality Number (by Fragment Analysis System).

## Data Availability

The data presented in this study are available within the article. If required, any additional data is available on request from the authors.

## Supplementary Materials

The following are available online at https://www.mdpi.com/2079-7737/10/3/209/s1, Figure S1: Area of stem-base cryosection recovered by laser microdissection given by the software. (A) LM software showing the harvested area. (B) Harvested phloem tissue corresponding to the area showed by the LM software, Figure S2: Cross section of the stem nodal segment of olive microcuttings at the site of adventitious root formation showing a region of the cortex being dislodged after laser cutting, Figure S3: Regions of the (A) epidermis (plus hypodermis), (B) cortex, and (C) phloem, completely separated from the stem-base cryosection, Figure S4: Cross sections of the stem basal segments of olive microcuttings at the site of adventitious root formation. (A) first cell divisions (Cd) at 6 days after rooting induction, leading to callus formation. (B) meristemoid structure (Me) in the upper phloem 10 days after rooting induction. (C) meristemoid structure (Me) in the cortex/sub-epidermal (hypodermis) region 10 days after rooting induction. (D) morphogenic root zones (Rf) developing from sub-epidermal (hypodermis) cells 14 days after rooting induction. Adapted from Macedo et al. [29]. Black circles: regions containing the structures/zones described above to be selected by LM. Blue circles: regions with no visible alterations to be used as control samples.
