# Erratum: Velada et al. Laser Microdissection of Specific Stem-Base Tissue Types from Olive Microcuttings for Isolation of High-Quality RNA. *Biology* 2021, *10*, 209

**DOI:** 10.3390/biology10080718

**Published:** 2021-07-28

**Authors:** Isabel Velada, Esther Menéndez, Rita Teresa Teixeira, Hélia Cardoso, Augusto Peixe

**Affiliations:** 1 MED—Mediterranean Institute for Agriculture, Environment and Development, Institute for Advanced Studies and Research, Universidade de Évora, Pólo da Mitra, Ap. 94, 7006-554 Évora, Portugal; esthermenendez@uevora.pt (E.M.); hcardoso@uevora.pt (H.C.); 2 BioISI—Biosystems & Integrative Sciences Institute, Faculty of Sciences, University of Lisbon, 1749-016 Lisbon, Portugal; rtteixeira@fc.ul.pt; 3 MED—Mediterranean Institute for Agriculture, Environment and Development and Departamento de Fitotecnia, Escola de Ciências e Tecnologia, Universidade de Évora, Pólo da Mitra, Ap. 94, 7006-554 Évora, Portugal; apeixe@uevora.pt

The author wishes to make an erratum to the published version of the paper [1]. 

In the original article, the funder “National Funds through FCT—Foundation for Science and Technology under the Project UIDB/05183/2020” was included under the Acknowledgments Section rather than the Funding Section.

It should be as follows:

**Funding:** This work is funded by National Funds through FCT—Foundation for Science and Technology under the Project UIDB/05183/2020.
**Acknowledgments:** This research was supported by the Project OLEAVALOR (ALT20-03-0145-FEDER-000014) supported by FEDER funds through the Program Alentejo 2020 and by the CALL ICAAM 2016 and 2017 (UID/AGR/00115/2013). I.V. was supported by the post-doc1_oleavalor fellowship (Project OLEAVALOR). E.M. was supported by a postdoctoral contract associated with the project UID/AGR/00115/2013. H.C. was supported by the FCT post-doc fellowship SFRH/BPD/109849/2015. The authors are grateful to Virgínia Sobral (Plant Breeding and Biotechnology Lab, University of Évora) for all technical assistance with the plant material.

The authors apologize for any inconvenience caused and state that the scientific conclusions are unaffected. The original article has been updated.
