# Supplementary material for: Laser Microdissection of Specific Stem-Base Tissue Types from Olive Microcuttings for Isolation of High-Quality RNA
Source: Biology (Basel). 2021 Mar 10;10(3):209. doi: 10.3390/biology10030209 (PMC7999021; doi:10.3390/biology10030209)
Supplement: Supplementary file 1 [file biology-10-00209-s001.pdf]

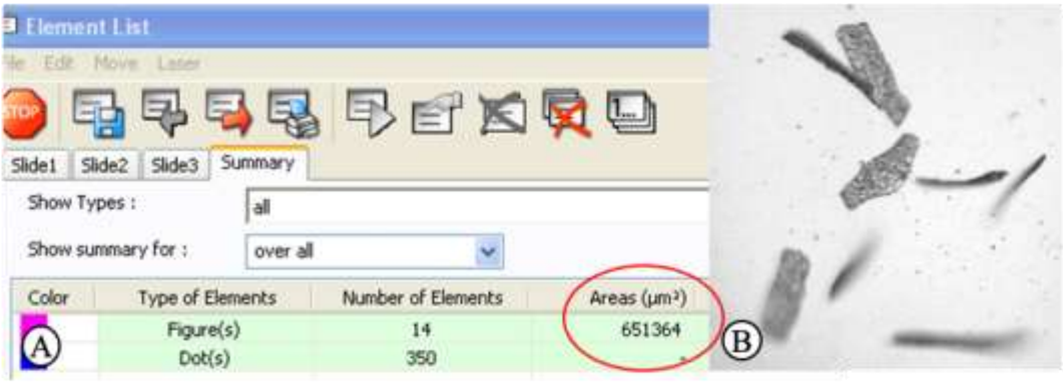

Figure S1 – Area of stem-base cryosection recovered by laser microdissection given by the software. A) LM software showing the harvested area. B) Harvested phloem tissue corresponding to the area showed by the LM software.

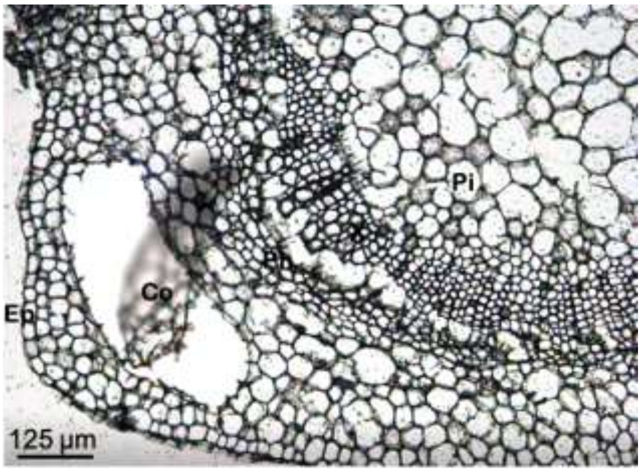

Figure S2 – Cross section of the stem nodal segment of olive microcuttings at the site of adventitious root formation showing a region of the cortex being dislodged after laser cutting.

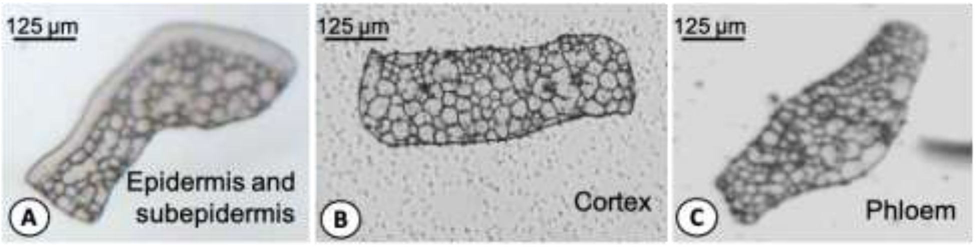

Figure S3 – Regions of the (A) epidermis (plus subepidermis), (B) cortex, and (C) phloem, completely separated from the stem-base cryosection.

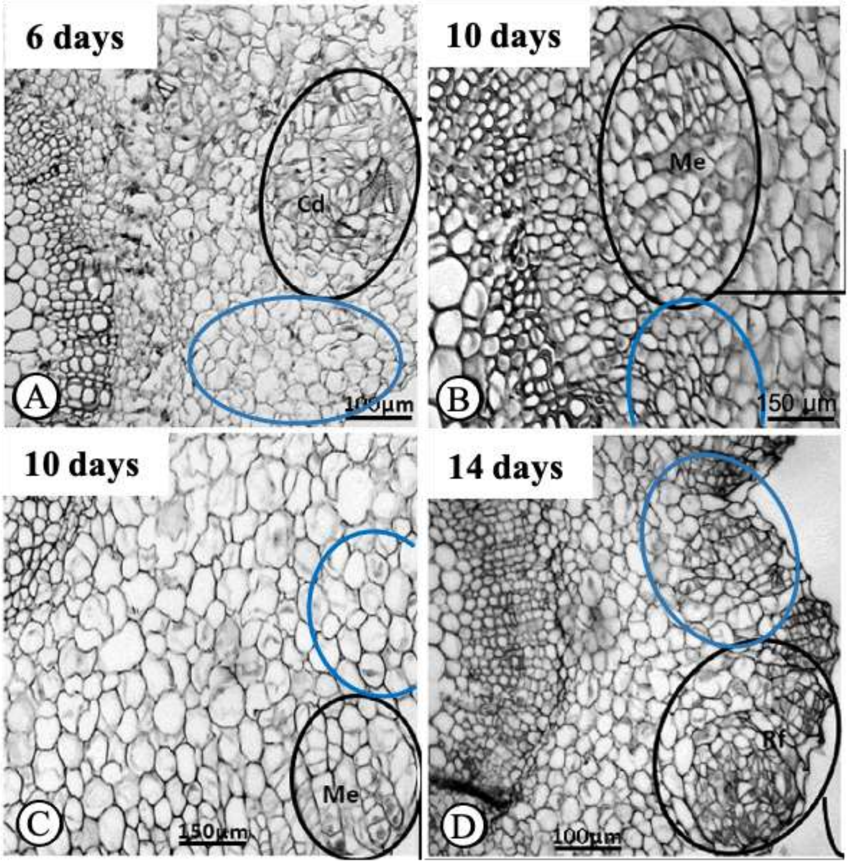

Figure S4 – Cross sections of the stem basal segments of olive microcuttings at the site of adventitious root formation. A) first cell divisions (Cd) at 6 days after rooting induction, leading to callus formation. B) meristemoid structure (Me) in the upper phloem 10 days after rooting induction. C) meristemoid structure (Me) in the cortex/sub-epidermal region 10 days after rooting induction. D) morphogenic root zones (Rf) developing from sub-epidermal cells 14 days after rooting induction. Adapted from Macedo et al [27]. Black circles: regions containing the structures/zones described above to be selected by LM. Blue circles: regions with no visible alterations to be used as control samples.
